# Supplementary material for: Micelle-like clusters in phase-separated Nanog condensates: A molecular simulation study
Source: PLoS Comput Biol. 2023 Jul 24;19(7):e1011321. doi: 10.1371/journal.pcbi.1011321 (PMC10399900; doi:10.1371/journal.pcbi.1011321)
Supplement: S1 Appendix — (DOCX) [file pcbi.1011321.s001.docx]

**S1 Appendix. The detail of the analysis**

**Micelle-like clusters in phase-separated Nanog condensates: A molecular simulation study**

Azuki Mizutani^1^, Cheng Tan^2^, Yuji Sugita^2, 3, 4^, and Shoji Takada^1^

^1^Department of Biophysics, Graduate School of Science, Kyoto University, Kyoto 606-8502, Japan, ^2^Computational Biophysics Research Team, RIKEN Center for Computational Science, 7-1-26 Minatojima-Minamimachi, Chuo-ku, Kobe, Hyogo 650-0047, Japan.

^3^Theoretical Molecular Science Laboratory, RIKEN Cluster for Pioneering Research, 2-1 Hirosawa, Wako, Saitama 351-0198, Japan.

^4^Laboratory for Biomolecular Function Simulation, RIKEN Center for Biosystems Dynamics Research, 1-6-5 Minatojima-Minamimachi, Chuo-ku, Kobe, Hyogo 650-0047, Japan.

**Preprocessing for the analysis of trajectories**

In each trajectory, we converted the coordinates of coarse-grained (CG) particles for later analysis using “crd_convert”, a tool in the GENESIS-1.7.1[1][2][3]. Then, we moved the center of mass of the system to the origin to fix the condensate at the center. Next, we corrected the position of amino acids so that all amino acids of one molecule are contiguous in the periodic boundary.

**Clustering and calculation of the concentration of Nanog in dilute phase**

To calculate the Nanog concentration in the dilute phase. We need to determine whether a molecule is in the dilute phase or not. To do so, for each snapshot, we first calculated the distance between every two molecules. The distance between the *i*-th and *j*-th molecules was defined as the minimum of the distances between the residues in the *i*-th and *j*-th molecules. Using the distance cutoff 50Å, we obtained the contact map. We then performed the clustering based on the contact map, and decided the molecules in the cluster which contained less than four molecules as those in the dilute phase.

**The convergence of each simulation**

To check the convergence of each simulation, we examined the change of the condensate state by the following two methods. 1) We defined the breadth of the condensate as the range of the Z-axis of the centroids of the molecules in the condensed phase. We first performed the clustering and decided the molecules in the condensed phase. In each time step, we estimated the breadth of the condensed phase and plotted it along the timestep (Fig. S2A). 2) We counted the number of molecules in the condensed phase. We performed the clustering based on the contact between the coarse-grained particles and counted the number of molecules (Fig. S2B). As shown in Figure S2A or S2B, both the breadth of the condensed phase and the number of molecules in the condensate changed in the first $\sim1\times{10}^{7}$ MD steps, but they did not change significantly after that. Thus, to remove the effect of initial structures we used data after $1\times{10}^{7}$MD step for the later analysis.

**The diffusion in the condensed phase**

To study the dynamics in the condensed phase, we calculated the mean-square deviation (MSD) of each Nanog molecule in the condensed phase and comparted it with that in the dilute phase. We assumed that the diffusion coefficient of a Nanog molecule in the surface or the interior of the condensed phase could be different.

To estimate the diffusion in the interior of the condensed phase, we calculated the number of inter-molecule contacts for each molecule in the condensed phase and defined the Nanog molecules interior of the condensed phase as those having 10 or more contacts with other Nanog molecules. For the interior Nanog molecules, we calculated the MSD as a function of the time difference (Fig. S4 or S5E).

**Effect of the globular domain**

As in the “Method” section, we applied the HPS potential only to NTD and CTD. This is because the parameters of the HPS potentials were tuned only for disordered regions of proteins[4][5]. To address effects of physicochemical interactions between globular domains and disordered domains, we examined a case that the HPS potential was applied to all the pairwise interactions including DBD. We performed a $3\times{10}^{7}$step simulation with the same simulation box and compared the result (Fig. S6). While no molecules diffused to the dilute phase during the simulation (Fig. S6A), the inter-molecule residue contact map obtained was similar to the case that the HPS potential was not applied to DBD (Fig. S6B). We found similar micelle-like clusters in the condensate (Fig. S6C). We suggested that the hydrophobic/hydrophilic interactions between DBD and N- or C-terminal domains did not significantly affect structures of the Nanog condensate.

Many previous studies about LLPS using coarse-grained models did not contain the globular domains[6][7][8]. To study the effect of globular domains on LLPS we made a model of a mutant that lacks the DBD. We performed a $3\times{10}^{7}$steps simulation with the similar setup and found that the phase-separated form was stable (Fig. S7A, B). Some molecules occasionally diffused to the dilute phase. The contact map was like the original one (Fig. S7C): the WRs interacted with WRs. Nanog formed micelle-like clusters (Fig. S7B)

Based on these results, we suggested that the globular domains did not significantly affect the structure of the Nanog condensate.

**Reference**

1. Jung J, Mori T, Kobayashi C, Matsunaga Y, Yoda T, Feig M, et al. GENESIS: A hybrid-parallel and multi-scale molecular dynamics simulator with enhanced sampling algorithms for biomolecular and cellular simulations. Wiley Interdiscip Rev Comput Mol Sci. 2015;5: 310–323. doi:10.1002/wcms.1220

2. Kobayashi C, Jung J, Matsunaga Y, Mori T, Ando T, Tamura K, et al. Genesis 1.1: A hybrid-parallel molecular dynamics simulator with enhanced sampling algorithms on multiple computational platforms. J Comput Chem. 2017;38: 2193–2206. doi:10.1002/jcc.24874

3. Tan C, Jung J, Kobayashi C, Torre DU La, Takada S, Sugita Y. Implementation of residue-level coarsegrained models in GENESIS for large-scale molecular dynamics simulations. PLoS Comput Biol. 2022;18: 1–30. doi:10.1371/journal.pcbi.1009578

4. Dignon GL, Zheng W, Kim YC, Best RB, Mittal J. Sequence determinants of protein phase behavior from a coarse-grained model. Ofran Y, editor. PLOS Comput Biol. 2018;14: e1005941. doi:10.1371/journal.pcbi.1005941

5. Tesei G, Schulze TK, Crehuet R, Lindorff-Larsen K. Accurate model of liquid-liquid phase behavior of intrinsically disordered proteins from optimization of single-chain properties. Proc Natl Acad Sci U S A. 2021;118. doi:10.1073/pnas.2111696118

6. Joseph JA, Reinhardt A, Aguirre A, Chew PY, Russell KO, Espinosa JR, et al. Physics-driven coarse-grained model for biomolecular phase separation with near-quantitative accuracy. Nat Comput Sci. 2021;1: 732–743. doi:10.1038/s43588-021-00155-3

7. Benayad Z, Von Bülow S, Stelzl LS, Hummer G. Simulation of FUS Protein Condensates with an Adapted Coarse-Grained Model. J Chem Theory Comput. 2021;17: 525–537. doi:10.1021/acs.jctc.0c01064

8. Regy RM, Thompson J, Kim YC, Mittal J. Improved coarse-grained model for studying sequence dependent phase separation of disordered proteins. Protein Sci. 2021;30: 1371–1379. doi:10.1002/pro.4094

9. Boija A, Klein IA, Sabari BR, Dall’Agnese A, Coffey EL, Zamudio A V., et al. Transcription Factors Activate Genes through the Phase-Separation Capacity of Their Activation Domains. Cell. 2018;175: 1842-1855.e16. doi:10.1016/j.cell.2018.10.042
