# Supplementary figures and images for: Micelle-like clusters in phase-separated Nanog condensates: A molecular simulation study
Source: PLoS Comput Biol. 2023 Jul 24;19(7):e1011321. doi: 10.1371/journal.pcbi.1011321 (PMC10399900; doi:10.1371/journal.pcbi.1011321)

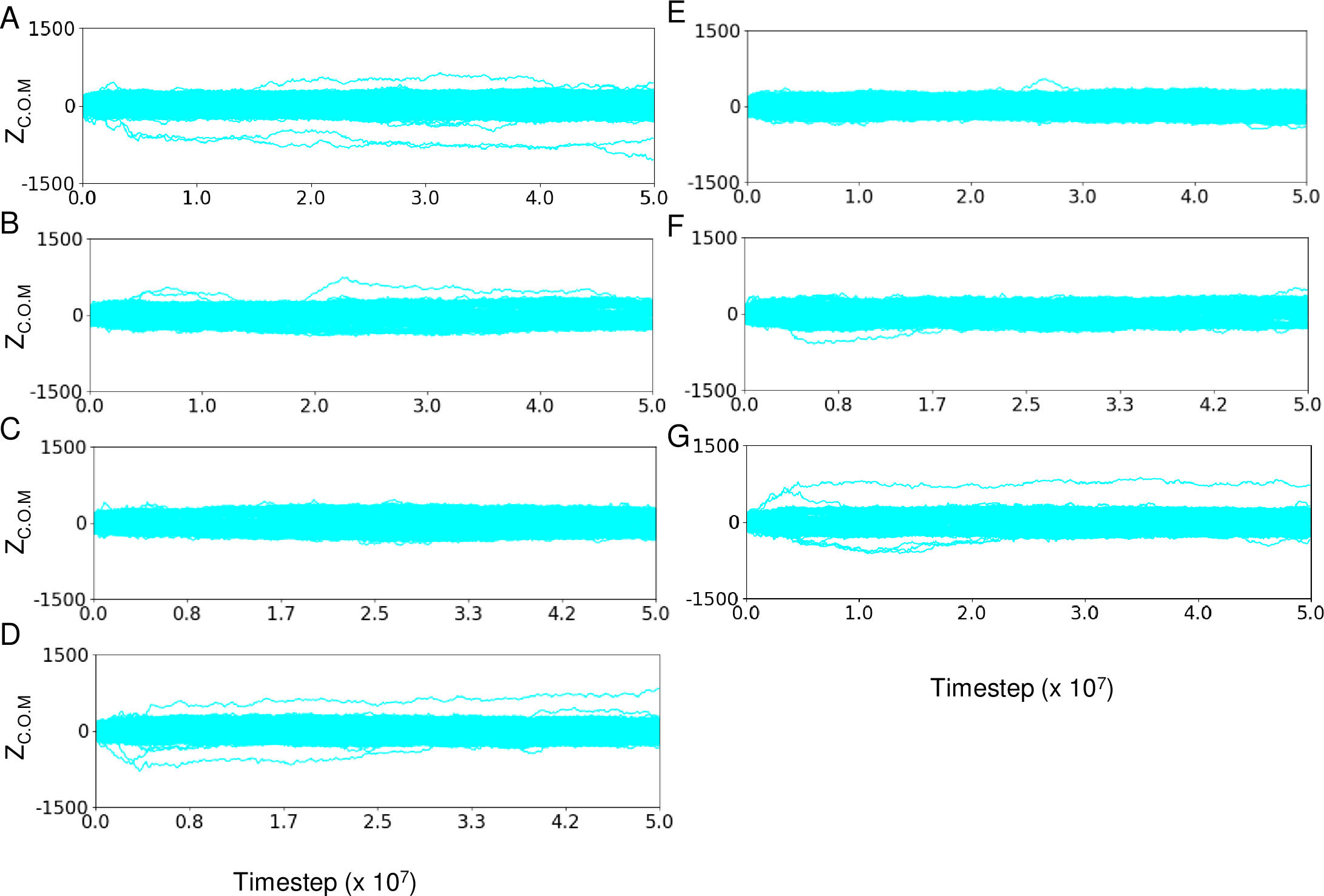

Supplement: S1 Fig — (A~G) The results of the MD simulation for 200 Nanog with the same setup as Fig 2 but with different random numbers. Cyan lines in each figure represent the trajectory of z-axis of centroids for each molecule during the simulation. (TIF) [file pcbi.1011321.s002.tif]

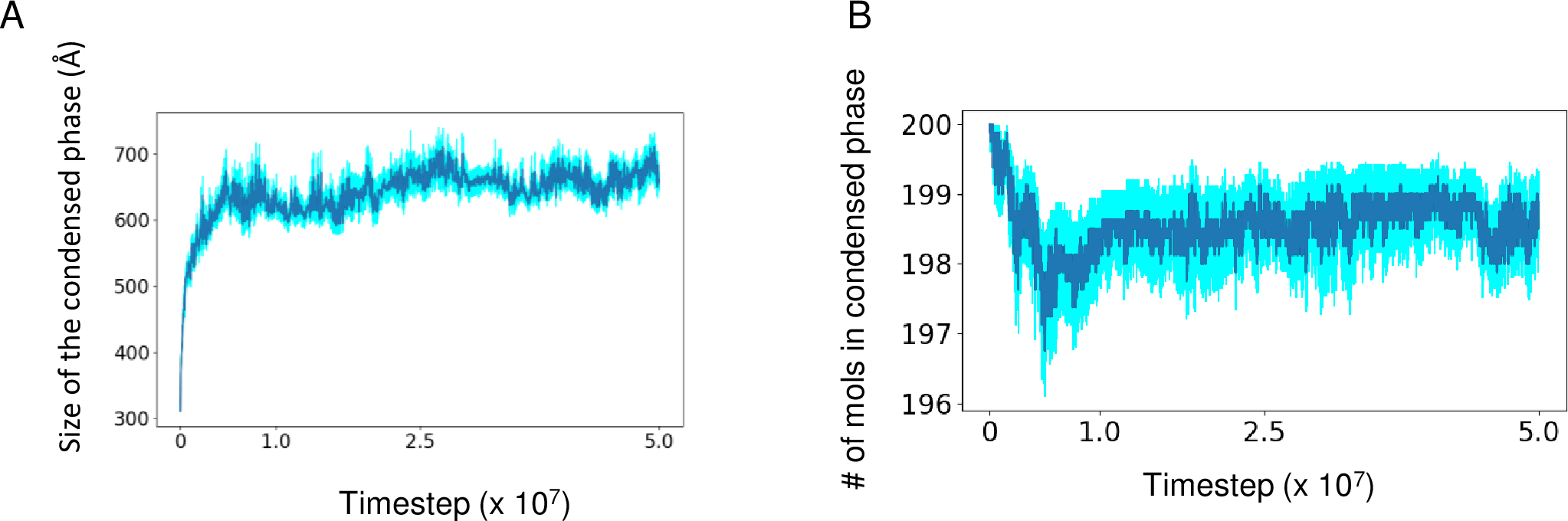

Supplement: S2 Fig — (A)The time course of the size of the condensed phase. (B)The time course of the number of molecules in the condensed phase. In (A) and (B), the blue curve represents the average of all the eight trajectories, and the cyan represents the standard error. (TIF) [file pcbi.1011321.s003.tif]

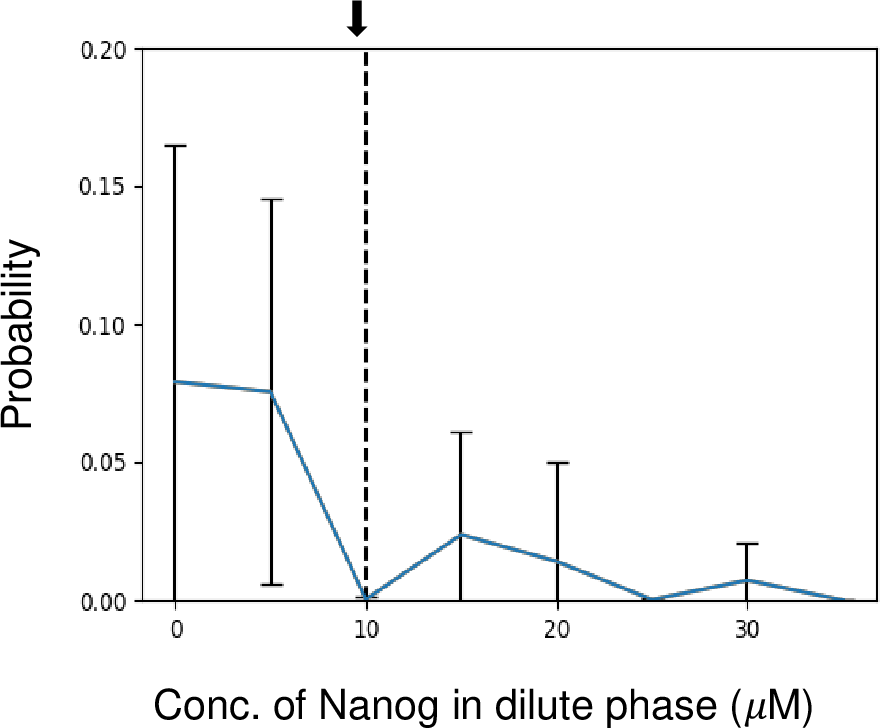

Supplement: S3 Fig — The distribution of the concentration of molecules in the dilute phase. The horizontal axis represents the concentration of the molecules in the dilute phase, calculated from the volume of the dilute phase and the number of molecules in the dilute phase. The distribution was calculated for each trajectory, which was then averaged. Each error bar represents the standard deviation in eight trajectories. The vertical dashed line represents the experimental upper limit of the critical concentration (10μM) [9]. (TIF) [file pcbi.1011321.s004.tif]

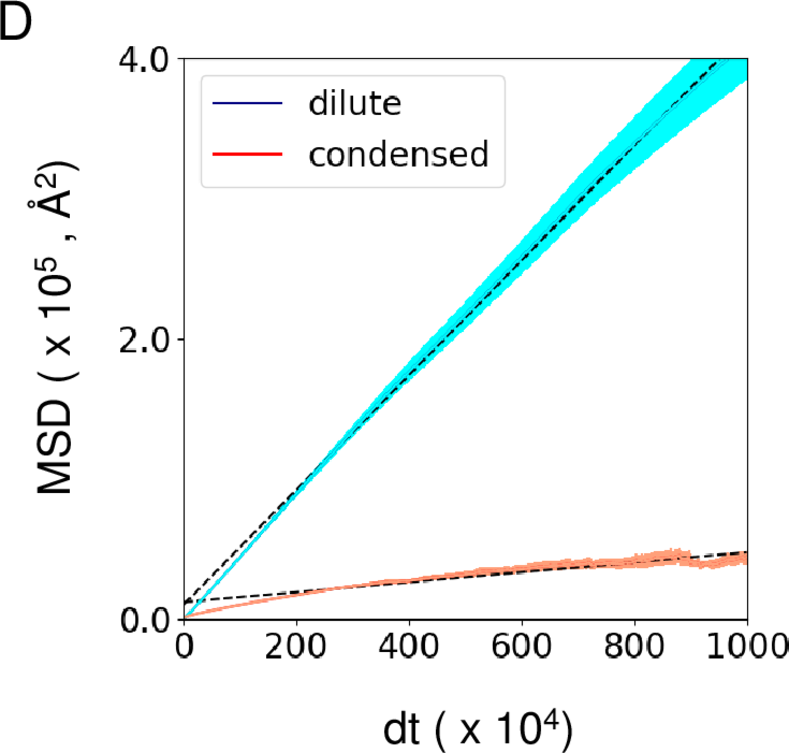

Supplement: S4 Fig — The mean square deviation (MSD) of a Nanog molecule as a function of the time difference. Comparison of the simulation results for a single Nanog molecule (orange) and the condensed state (cyan). Filled regions represent the standard deviation in all the eight trajectories. The dashed lines are obtained by the liner regression. (TIF) [file pcbi.1011321.s005.tif]

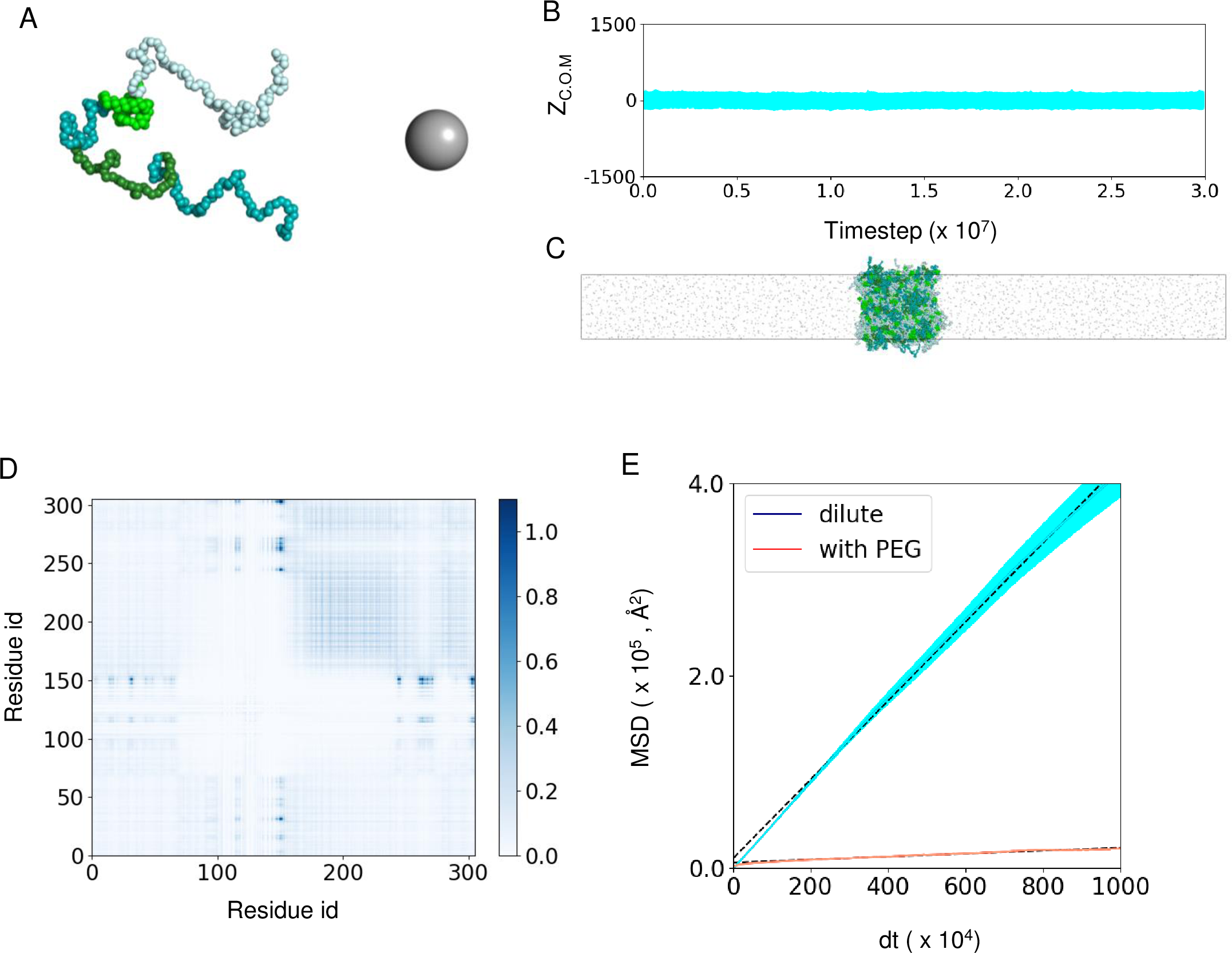

Supplement: S5 Fig — (A) The model of human Nanog and the PEG8000. The model of Nanog is the same as that in Fig 1C. NTD, DBD, CTD, WR are colored with palecyan, green, teal, forest. The gray sphere represents a model for one molecule of PEG8000. (B) The result of one trajectory. Cyan curves represent the trajectories of z-axis of the centroids of 200 Nanog molecules. (C) The snapshot at the last frame of the same trajectory. We drew PEG8000 spheres with the radius smaller than the actual one. (D) The inter-molecule contact map. We used the cutoff distance 6.5Å between the two residues. The color represents the average number of contacts over all the frames and all trajectories. (E) The MSD as a function of the time difference. The yellow line represents the MSD of Nanog in the condensed phase with PEG. The blue line is the same result as in S4 Fig. (TIF) [file pcbi.1011321.s006.tif]

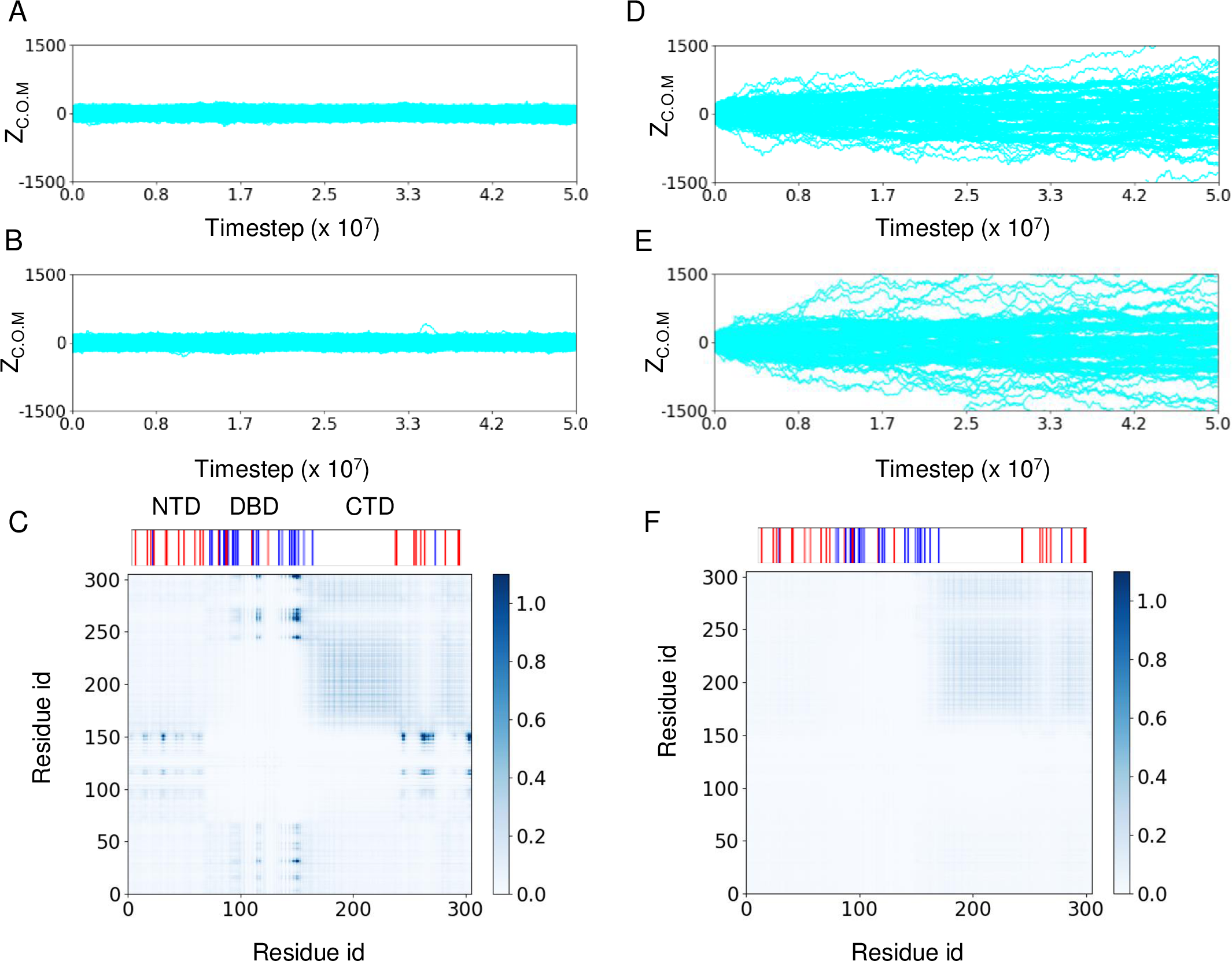

Supplement: S6 Fig — (A, B) Time courses of 200 Nanog molecules along the z-axis (long axis of the slab) in the simulations with 50 mM condition. Fig A and B are results of the same setup with different random number seeds. (C) The residue contact map between two Nanog molecules in the simulation with 50 mM conditions. (D, E) Time courses of 200 Nanog molecules along the z-axis (long axis of the slab) in the simulations with 500 mM condition. Fig C and D are results of the same setup with different random number seeds. (F) The residue contact map between two Nanog molecules in the simulation with 500 mM NaCl conditions. Residue pairs of different molecules within 6.5Å are considered as the contact in (C) and (F). (TIF) [file pcbi.1011321.s007.tif]

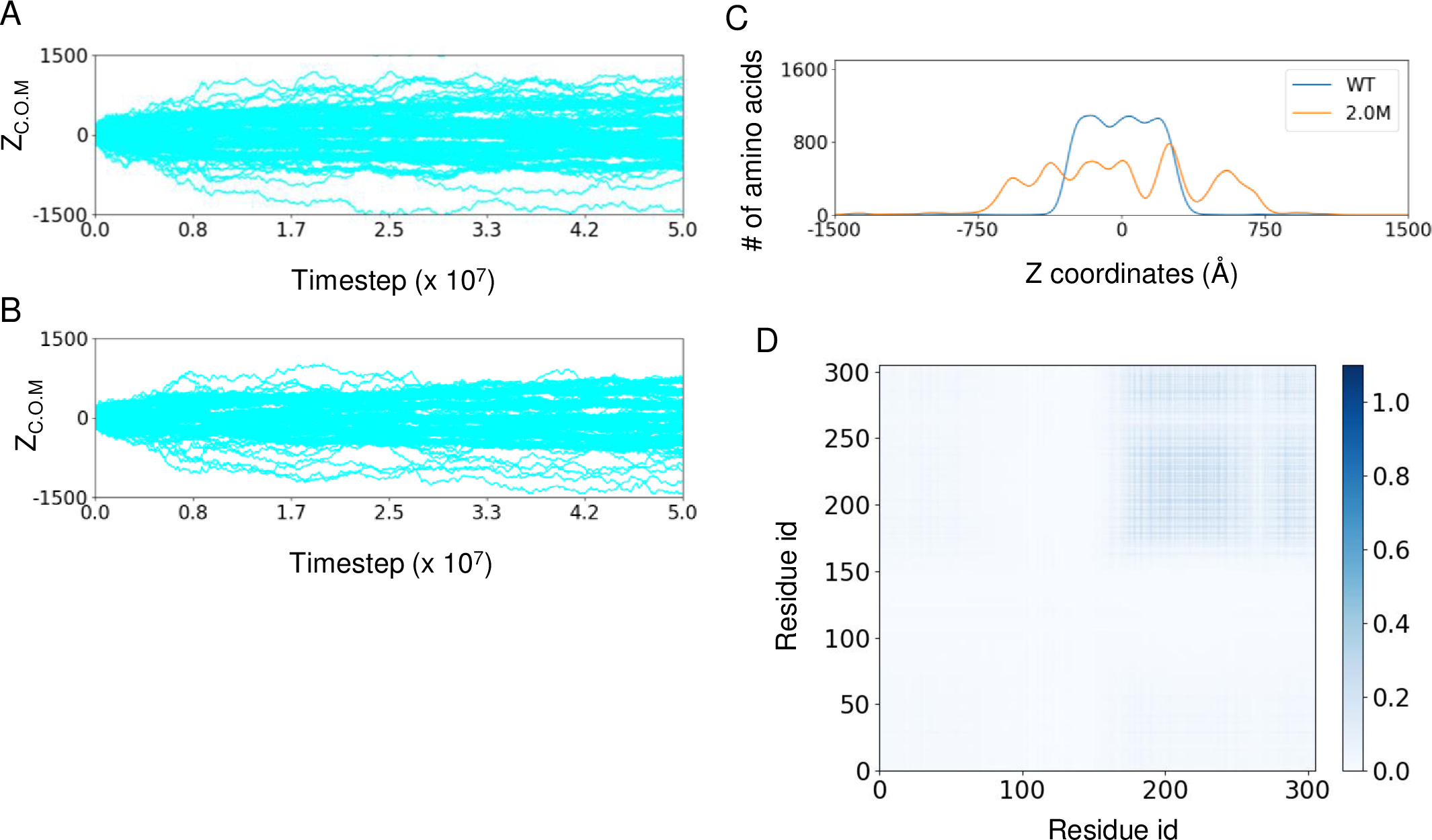

Supplement: S7 Fig — (A, B) Time courses of 200 Nanog molecules along the z-axis (long axis of the slab) in the simulations with 2.0 M condition. Fig A and B are results of the same setup with different random number seeds. (C) The distribution of Nanog amino acids along the z-axis; Blue: WT, Yellow: with 2.0M condition. (D) The residue contact map between two Nanog molecules. Residue pairs of different molecules within 6.5Å are considered as the contact. (TIF) [file pcbi.1011321.s008.tif]

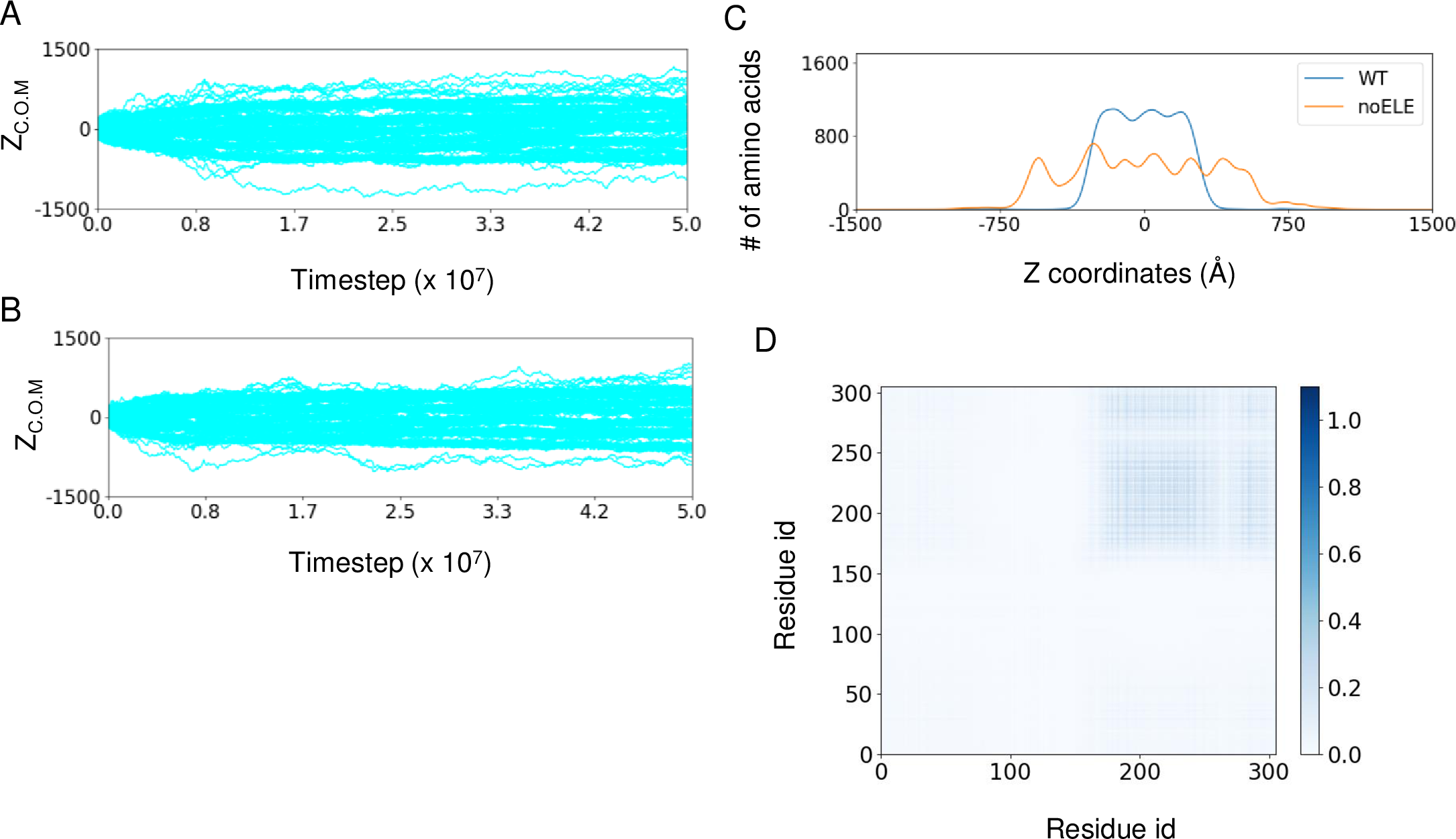

Supplement: S8 Fig — (A, B) Time courses of 200 Nanog molecules along the z-axis (long axis of the slab) in the simulations without the electrostatic interaction. Fig A and B are results of the same setup with different random number seeds. (C) The distribution of Nanog amino acids along the z-axis; Blue: WT, Yellow: without the electrostatic interaction. (D) The residue contact map between two Nanog molecules. Residue pairs of different molecules within 6.5Å are considered as the contact. (TIF) [file pcbi.1011321.s009.tif]

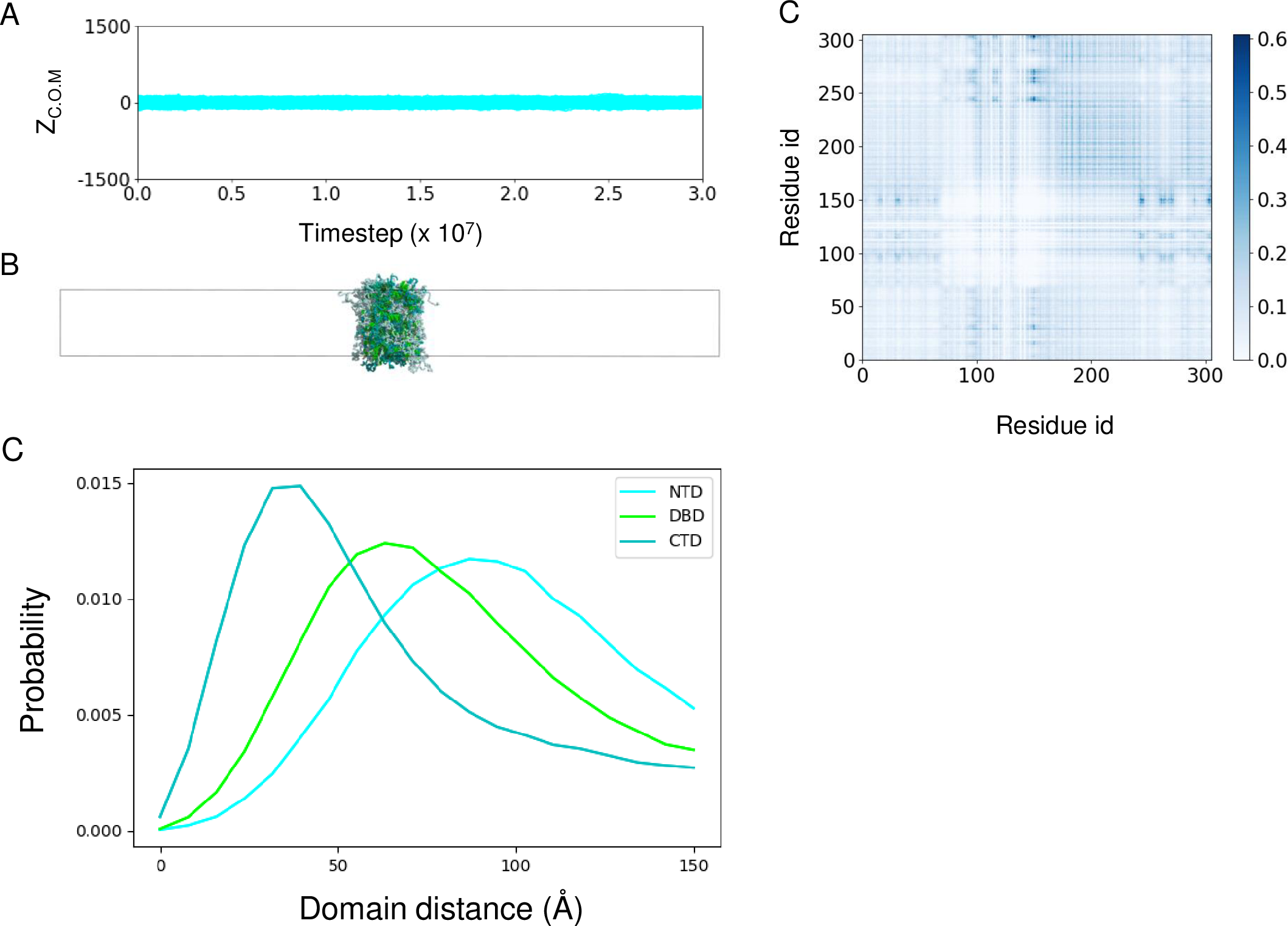

Supplement: S9 Fig — (A) Time courses of 200 Nanog molecules along the z-axis (long axis) of the slab. (B) The snapshot at the end of the same simulation as in A. (C) The residue contact map between two Nanog molecules. Residue pairs of different molecules within 6.5 Å are considered as the contact. The averaged numbers of contacts in all frames and trajectories are depicted. (D) The distributions of the distances of each domain from the center of mass of the cluster. Cyan, NTD; green, DBD; teal, CTD. (TIF) [file pcbi.1011321.s010.tif]

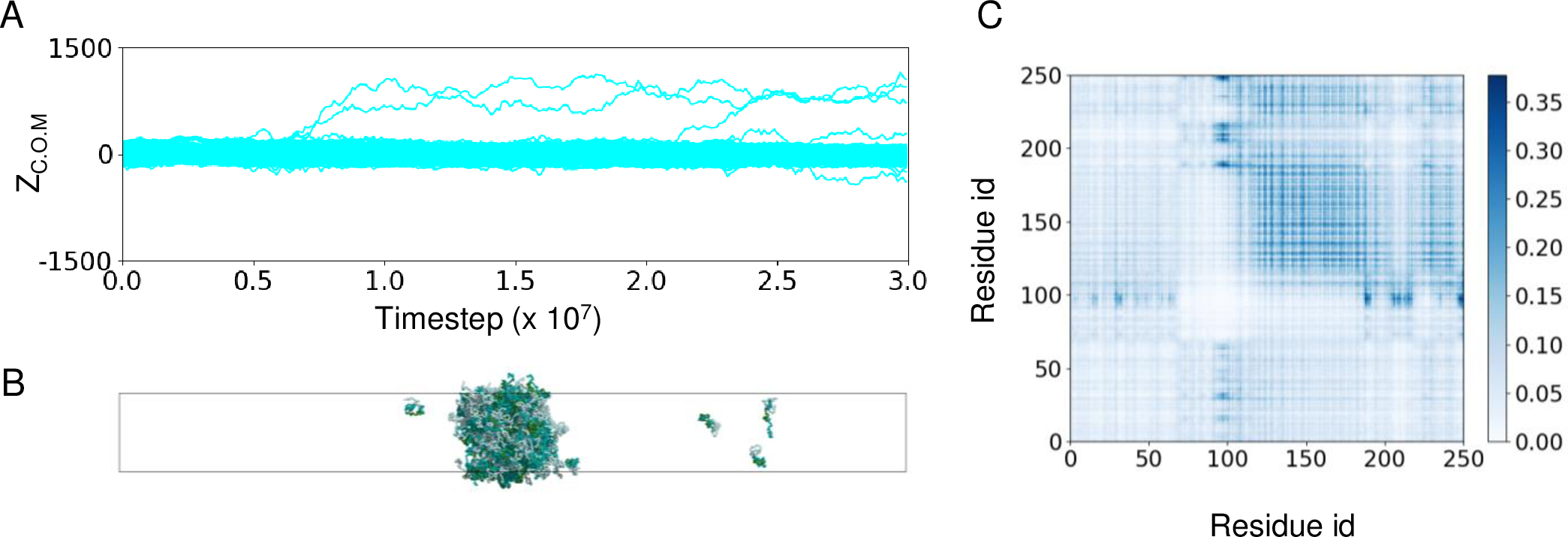

Supplement: S10 Fig — (A) Time courses of 200 mutant molecules along the z-axis of the slab. (B) The snapshot at the end of the same simulation in A. (C) The residue contact map between two Nanog molecules. Residue pairs of different molecules within 6.5 Å are considered as the contact. The averaged numbers of contacts in all frames and trajectories are depicted. (TIF) [file pcbi.1011321.s011.tif]

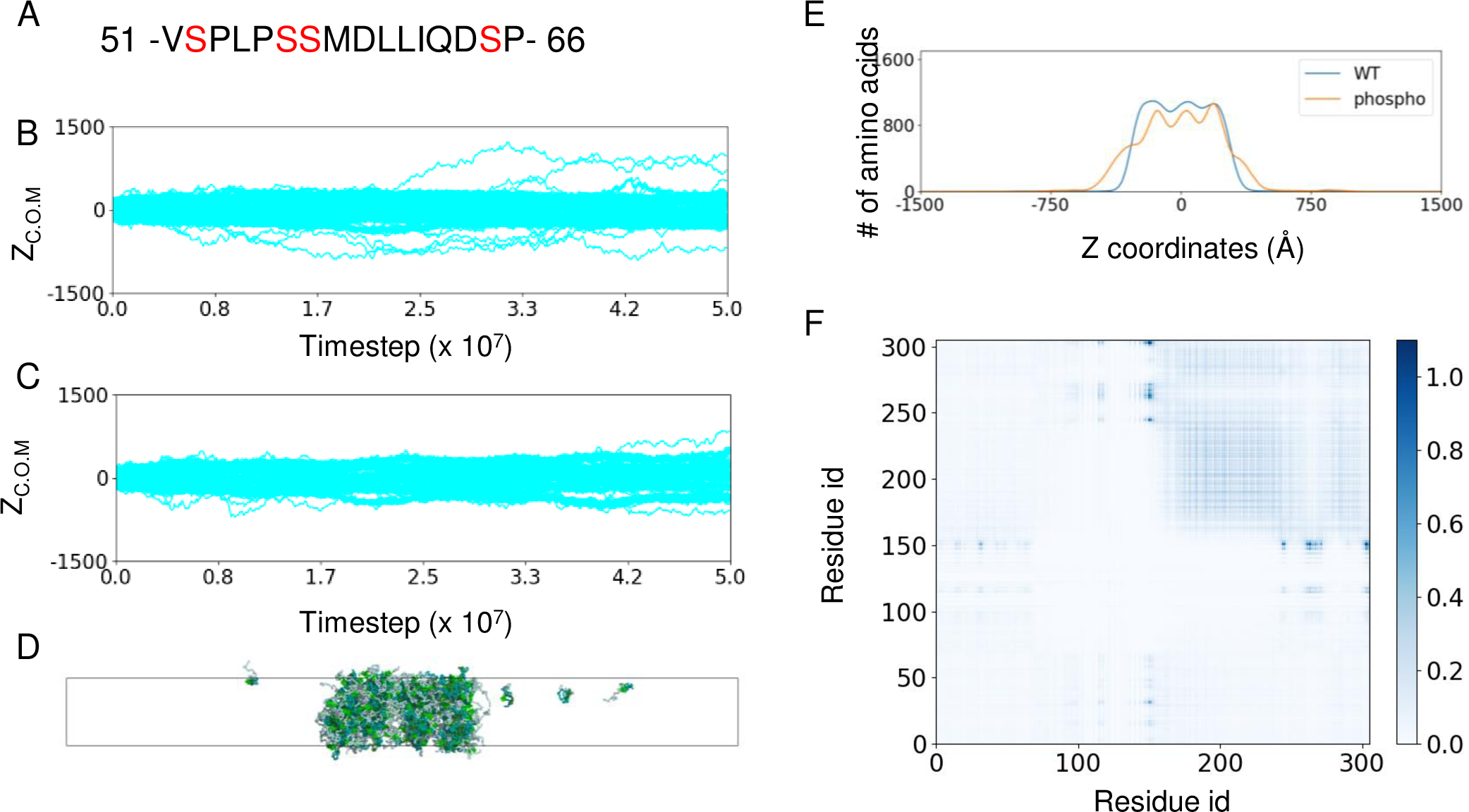

Supplement: S11 Fig — (A)The mutated residues in the N-terminal domain of Nanog. The red colored serine were phosphorylated to have the charges -1.0. (B, C) Time courses of 200 Nanog mutants along the Z-axis (long axis) of the slab. Fig B and C represent results of the same setup with different random number seeds. (D) Snapshot of the end of the same simulations as shown in Fig A. (E) Density of amino acids along the z-axis. yellow, phosphorylated; blue, WT. (F) The residue contact map between two phosphorylated Nanog molecules in the simulation. Residue pairs of different molecules within 6.5Å are considered as the contact. (TIF) [file pcbi.1011321.s012.tif]

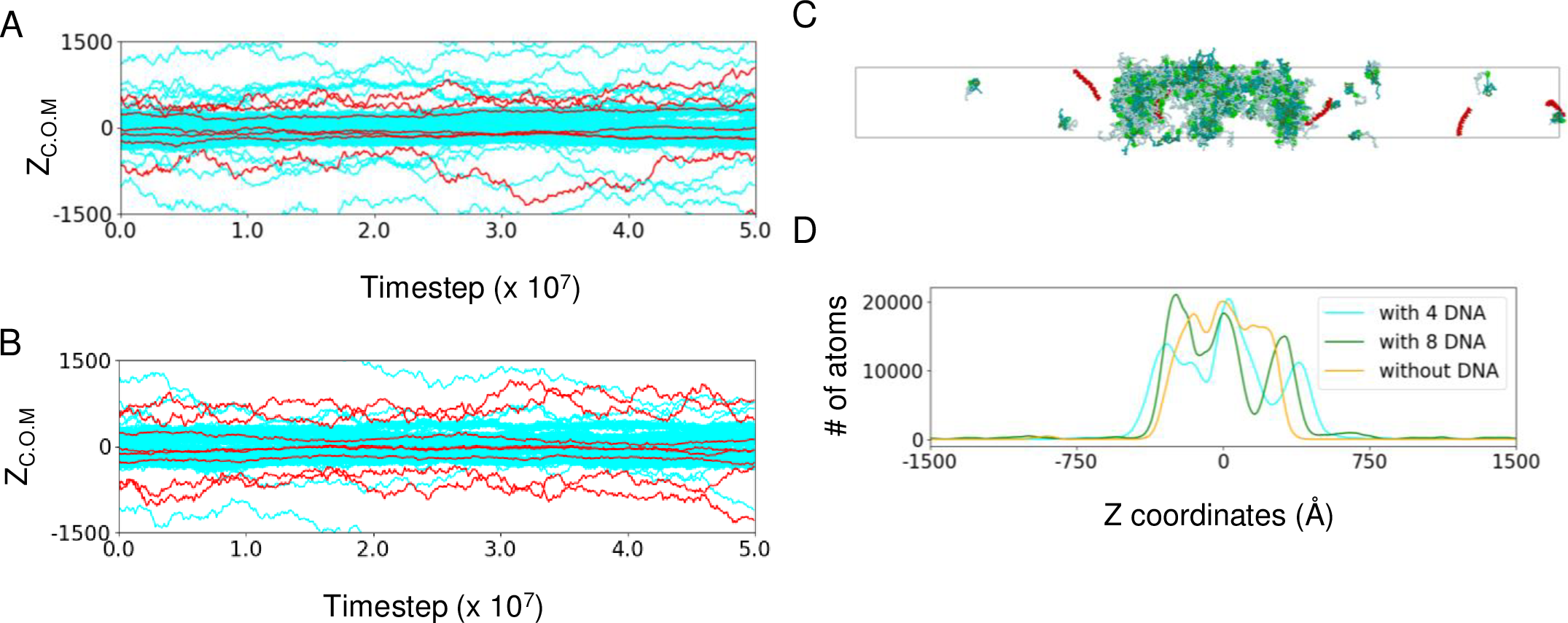

Supplement: S12 Fig — (A, B) Time courses of 200 Nanog molecules (cyan) and eight DNA (red) along the z-axis (long axis) of the slab. (C) Snapshot at the end of the same simulation as (A). (D) Atomic density calculated from each type of CG particles including Nanog and DNA along the z-axis in the simulations of 200 Nanog without DNA and with four or eight DNA fragments. (Blue, with four DNA; Green, with eight DNA; Orange, without DNA) (TIF) [file pcbi.1011321.s013.tif]
